# Healthy cardiac myocytes can decrease sympathetic hyperexcitability in the early stages of hypertension

**DOI:** 10.3389/fnsyn.2022.949150

**Published:** 2022-08-04

**Authors:** Harvey Davis, Kun Liu, Ni Li, Dan Li, David J. Paterson

**Affiliations:** ^1^Burson Sanderson Cardiac Science Centre, Department of Physiology, Anatomy & Genetics, University of Oxford, Oxford, United Kingdom; ^2^Department of Neuroscience, Physiology & Pharmacology, University College London, London, United Kingdom

**Keywords:** SHR (spontaneous hypertensive rat), cardiac excitability, sympathetic excitation, current clamp, dysautonomia

## Abstract

Sympathetic neurons are powerful drivers of cardiac excitability. In the early stages of hypertension, sympathetic hyperactivity is underpinned by down regulation of M current and increased activity of Cav_2.2_ that is associated with greater intracellular calcium transients and enhanced neurotransmission. Emerging evidence suggests that retrograde signaling from the myocyte itself can modulate synaptic plasticity. Here we tested the hypothesis that cross culturing healthy myocytes onto diseased stellate neurons could influence sympathetic excitability. We employed neuronal mono-cultures, co-cultures of neonatal ventricular myocytes and sympathetic stellate neurons, and mono-cultures of sympathetic neurons with media conditioned by myocytes from normal (Wistar) and pre-hypertensive (SHR) rats, which have heightened sympathetic responsiveness. Neuronal firing properties were measured by current-clamp as a proxy for neuronal excitability. SHR neurons had a maximum higher firing rate, and reduced rheobase compared to Wistar neurons. There was no difference in firing rate or other biophysical properties in Wistar neurons when they were co-cultured with healthy myocytes. However, the firing rate decreased, phenocopying the Wistar response when either healthy myocytes or media in which healthy myocytes were grown was cross-cultured with SHR neurons. This supports the idea of a paracrine signaling pathway from the healthy myocyte to the diseased neuron, which can act as a modulator of sympathetic excitability.

## Introduction

Enhanced activity of the sympathetic nervous system is a common feature and early indicator of many cardiovascular diseases including heart failure, post-myocardial infarction and hypertension (Ajijola et al., 2015; Grassi et al., 2015; Schwartz et al., 2015; Habecker et al., 2016; Herring et al., 2019). In hypertension, this neural phenotype precedes the overt clinical signs of high blood pressure (Dominiak and Grobecker, 1982; Anderson et al., 1989) and contributes to the progression of the disease itself by facilitating other co-morbidities such as arrhythmia (Lown and Verrier, 1976), cardiac hypertrophy (Levick et al., 2010), inflammation (Singh et al., 2014), and end-organ damage (Julius et al., 1989). Superimposed on emerging evidence for structural and functional plasticity of the local circuit neurons in the spontaneously hypertensive rat (SHR) (Ashton et al., 2020), adds further support to the notion that the nervous system is an important driver and therapeutic target, even though the neurobiological basis of dysautonomia is still relatively poorly understood.

In the early stages of hypertension in the SHR, the heightened sympathetic responsiveness may result from down-regulation of M-current that restricts neural firing (Davis et al., 2020), local inflammation associated with macrophages (Neely et al., 2022), increased activity of Cav_2.2_ (Larsen et al., 2016a), impaired NO-cGMP signaling (Li et al., 2007), and abnormal regulation of mitochondrial phosphodiesterases (Li et al., 2022). These responses are linked to greater intracellular calcium (Ca^2+^) transients (Li et al., 2012; Neely et al., 2022) and facilitated exocytosis of classical transmitters (Lu et al., 2015) and neuropeptides (Herring et al., 2008). Combined with a slow re-uptake of norepinephrine by the noradrenaline-transporter (NET) (Shanks et al., 2013), the post-ganglionic pre-synaptic neuron appears to be a powerful driver of myocyte function. Moreover, it is well established in the SHR that a diseased Ca^2+^ phenotype also resides in the myocytes (Heaton et al., 2006), where recent evidence suggests that retrograde signaling from the myocyte itself might modulate synaptic plasticity (Habecker et al., 2016; Prando et al., 2018; Franzoso et al., 2022).

To test the hypothesis that the myocytes themselves may provide an important paracrine signal to modulate sympathetic excitability, we therefore examined whether cross-culturing healthy myocytes onto diseased stellate neurons could influence the activity of the neurons. Here we show that healthy myocytes or media in which they were grown when cross-cultured with SHR neurons could decrease neuronal excitability, supporting the idea of paracrine signaling from the myocyte to the neuron during the early phase of dysautonomia associated hypertension.

## Method

### Animals

Animal use complied with the University of Oxford Local Ethical Guidelines and was in accordance with the Guide for the Care and Use of Laboratory Animals published by the US National Institutes of Health (Publication No. 85-23, revised 2011) and the Animals (Scientific Procedures) Act 1986 (United Kingdom). Experiments were performed under British Home Office Project License [PPL 30/3131 (DP) and P707EB251 (DP)]. Male Wistar and SHR were purchased from Envigo, United Kingdom and housed in the local Biomedical Services Building on a 12-h day-night cycle prior to experimental use. Rats aged 5–6 weeks were euthanized via an overdose of pentobarbitone and confirmed via exsanguination according to Schedule 1 of the Animals (Scientific Procedures) Act 1986 (United Kingdom). At this age the SHR exhibits dysautonomia, but has not yet developed an increase in arterial blood pressure to act as a confounding variable (Minami et al., 1989; Dickhout and Lee, 1998; Li et al., 2012).

### Cell isolation and culturing

Stellate ganglia were dissected and immediately transferred to ice-cold HEPES buffered L15 media (L1518, Sigma Aldrich, United States). Ganglia were cut into 2 mm sections and enzymatically digested at 37°C first using 1 mg/ml Collagenase IV (Worthington, United States) in L15 for 25 min, followed by 30 min in 2 mg/ml Trypsin (Worthington, United States) in Ca^2+^ and Mg^2+^ free Hanks buffered salt solution (Thermo Fisher Scientific, United States). Enzymes were then inhibited using two washes of a blocking solution containing 10% FBS. The tissue was then suspended in a plating media described in Table 1 and mechanically disrupted using a fire-blown glass pipette. The cell suspension was then plated onto Poly-D-lysine coated Fluorodish 35 mm dishes (WPI, United States), which had been previously incubated for 2 h with 1 μg/ml laminin, a concentration chosen to allow cell adhesion and survival, but limiting neurite outgrowth. The cells were then incubated at 37°C with 5% CO_2_ for a period of 1–5 days *in vitro* before use. All datasets were recorded from at least two cultures, with each culture requiring four animals. Phase contrast microscopy was used to enable neuronal identification. Neurons were identified in dissociated culture based on their large size and circular somata relative to the surrounding cell types. Each culture was produced from a minimum of two animals, with each experiment being performed on a minimum of two cultures.

**TABLE 1:** L-15 based plating media. pH adjusted to 7.6 with HCl.

Components	Source	Amount
L-15 media	L1518, Sigma-Aldrich, United States	90 ml
24 mM NaHCO3	Sigma-Aldrich, United States	201.6 mg
38 mM Glucose	Sigma-Aldrich, United States	684 mg
10,000U/mL Penicillin-Streptomycin	Thermo Fisher Scientific, United States	0.5 ml
100μg/ml 2.5s NGF	Merck Millipore, United States	50 μl
Fetal bovine serum	Thermo Fisher Scientific, United States	10 ml

### Co-culture

Briefly, neonatal cardiomyocytes were dissociated from 1to 2 days old Wistar ventricles. Cells were cultured in M1 media (Table 2) for a period of 1 day, before a subsequent day in M2 media (Table 2). On the third day of culturing, media was exchanged for a 50:50 mix of M2 media and plating media. Where stated Wistar or SHR neurons were dissociated from 5 to 6-week-old rats as described for neurons alone, and then plated onto the cardiomyocyte layer and cultured for a further 24 h.

**TABLE 2:** Media for co-culture.

Components	Source	Amount	
		Medium 1	Medium 2
DMEM with 25mM HEPES	42430025, Invitrogen, United States	67 ml	75 ml
Medium 199	31150022, Invitrogen, United States	17.5 ml	17 ml
Horse serum	Thermo Fisher Scientific, United States	10 ml	5 ml
Newborn calf serum	Thermo Fisher Scientific, United States	5 ml	0.5 ml
200 mM Glutamine	Thermo Fisher Scientific, United States	1 ml	1 ml
10,000 U/mL Penicillin-streptomycin	Thermo Fisher Scientific, United States	100 μl	100 μl

### Electrophysiological data acquisition

All electrophysiological data were acquired using Winwcp (Version 5.4.0) and recorded via a Multiclamp 700B amplifier (Molecular Devices, United States) with an axon digi data 1550A (Molecular devices, United States) digitizer. All current clamp recordings were sampled at 10 kHz. M-current deactivation curves were sampled at 10 KHz. Perfusion Cells were constantly perfused at a rate of 5–6 ml/min, drugs were applied via this perfusion system. The external recording solution for recordings was as follows: 5.2 mM KCl, 140 mM NaCl, 1 mM MgCl_2_, 1.8 mM CaCl_2_, 10 mM HEPES, and 10 mM Dglucose. External solution pH was adjusted to 7.4 with NaOH. The internal solution was composed as follows: 130 mM K+ -Gluconate, 10 mM KCl, 10 mM HEPES, 10 mM Na+ -Phosphocreatine, 4 mM MgATP, 0.3 mM Na2GTP. Internal pH was adjusted to 7.3 with KOH. Recordings were performed at room temperature.

### Whole cell patch-clamp recordings

Whole-cell action potential recordings with Rs values >12 MΩ discarded. Current clamp recordings were bridge balanced and membrane potentials were corrected for liquid junction potentials. All recordings were monitored throughout, and recordings with RS changes >20% were discarded. Single action potentials were evoked via a 10 ms positive current injection, at the minimal required injection size.

### Statistical analysis

All statistical analyses were performed using Graphpad Prism 8 software. All data analyzed in this publication were treated as continuous. Data normality was examined using Anderson-Darling, D’Agostino and Pearson, Shapiro–Wilk, and Kolmogorov–Smirnov tests, and parametric or non-parametric tests selected based on the outcome of these. For statistical comparison Welch’s *t*-tests (parametric), Kruskal–Wallis (non-parametric) or Mann–Whitney tests (non-parametric) were employed. Results were considered statistically significant where *p* < 0.05.

## Results

### Pre-hypertensive SHR neurons exhibit increased excitability

**FIGURE 1:**
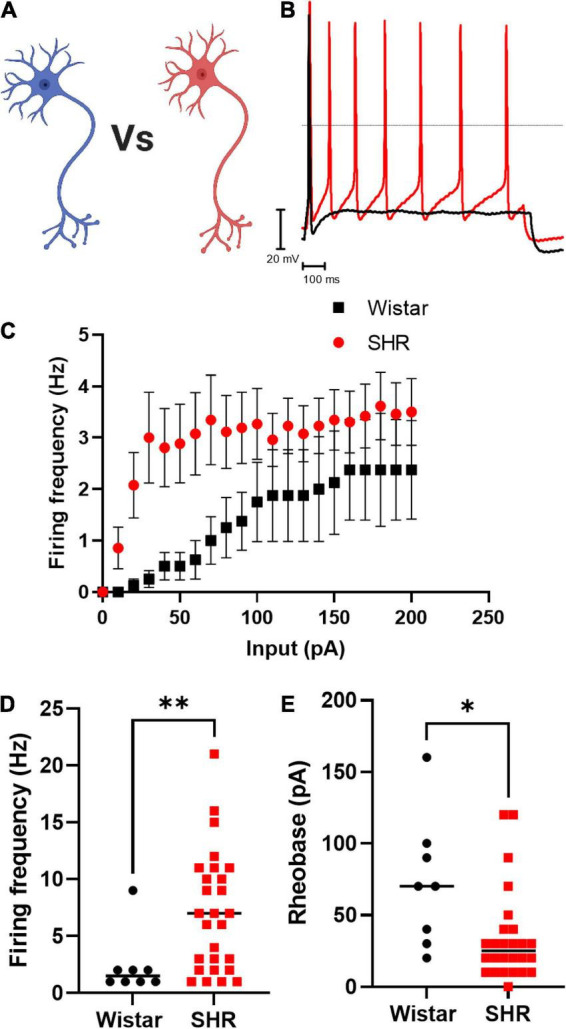
Primary cultured SHR neurons are more excitable than Wistar neurons Wistar and SHR neurons were cultured alone for a period of 3 days, and their intrinsic electrical excitability measured by whole-cell patch-clamp recordings of neuronal firing rate and rheobase. SHR neurons exhibited a greater firing rate, and a lower rheobase, both indicators of neuronal excitability. **(A)** Diagram of the experimental design in figure, where Wistar and SHR neurons cultured alone are compored by electrophysiological recordings. **(B)** Example traces of Wistar and SHR neuron firing induced by current injection are shown demonstrating the higher firing rate in SHR neurons. **(C)** The average (Mean) firing rate of Wistar and SHR neurons is shown for Wistar and SHR neurons. **(D)** The maximum firing frequency of SHR neurons when stimulated with 0–200 pA of current was shown to be greater than in Wistar neurons (Medians) (Mann–Whitney test; *n* = 8, *n* = 27; *p* = 0.0068). **(E)** The rheobase of SHR neurons is reduced relative to Wistar neurons, indicating a smaller current was required to induce neuronal firing (Medians) (Mann–Whitney test; *n* = 8, *n* = 26; *p* = 0.0113).

We first confirmed that Wistar and SHR neurons alone, exhibited hyperexcitability as measured by current-clamp whole-cell recordings (Figure 1). These experiments confirmed that the results obtained by our group previously (Davis et al., 2020) were reproducible in the media used here, and that the hyperexcitable phenotype was also observable in whole-cell recordings (compared to the perforated patch-clamp recordings used previously). We observed that the chosen range of current injections (0–200 pA) lead to a plateau in neuronal firing in both phenotypes, and that SHR neurons had a higher maximum firing rate (Figures 1C,D; Mann–Whitney test; *n* = 8, *n* = 27, *p* = 0.0068) and a reduced rheobase (Figure 1E; Mann–Whitney test; *n* = 8, *n* = 26, *p* = 0.0113).

### Cross culturing healthy myocytes onto diseased neurons decreases neuronal firing

**FIGURE 2:**
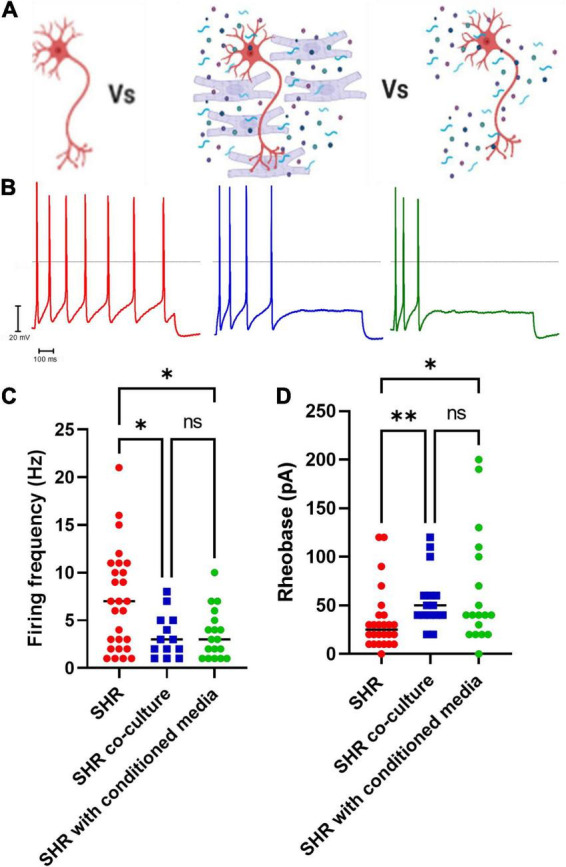
Co-culture of SHR neurons with Wistar cardiomyocytes or Wistar cardiomyocyte conditioned media reduces SHR neuron excitability. Wistar and SHR neurons were co-cultured with Wistar cardiomyocytes or with Wistar cardiomyocyte conditioned media, and neuronal intrinsic electrical excitability was measured by whole-cell patch-clamp recordings of neuronal firing rate and rheobase. SHR neurons cultured alone exhibited a greater firing rate, and a lower rheobase, than SHR neurons co-cultured with Wistar cardiomyocytes. **(A)** Diagram demonstrating the experimental design. Here SHR neurons (Red) were cultured with Wistar cardiomyocytes (Blue), and thus able to physically and/or chemically interact with the cardiomyocytes. Alternatively they were cultured with Wistar cardiomyocyte conditioned media (Blue), which was expected to contain any factor released by cardiomyocytes. **(B)** Example traces of SHR neurons cultured alone (Red), with wistar cardiomyocytes (Blue) or with cardiomyocyte conditioned media (Green). **(C)** The maximum firing frequency of SHR neurons cultured with cardiomyocytes (Blue) when stimulated with 0–200 pA of current was lower than in SHR neurons cultured alone (Red) (Medians) (Kruskall–Wallis test; *p* = 0.0281; Dunn’s uncorrected comparison; *n* = 27, *n* = 13, *p* = 0.0345). A similar result was observed between SHR neurons cultured alone (Red) or with Wistar cardiomyocyte conditioned media (Green) (Uncorrected Dunn’s test; *n* = 27, *n* = 18, *p* = 0.0222). No signficant difference was observed between SHR neurons cultured with wistar cardiomyocytes (Blue) or wistar cardiomyocyte conditioned culture media (Green) (Uncorrected Dunn’s test; *n* = 13, *n* = 18, *p* = 0.9617). **(D)** The rheobase of SHR neurons cultured with cardiomyocytes (Blue) was higher than in SHR neurons cultured alone (Red), indicating a greater current was required to induce neuronal firing (Medians) (Kruskal–Wallis test; *p* = 0.0071) (Uncorrected Dunn’s test; *n* = 0.0041). This was also observed for SHR neurons cultured alone (Red) or co-cultured with Wistar cardiomyocyte conditioned media (Green) (Uncorrected Dunn’s test; *n* = 0.0291). No difference was observed between SHR neurons co-cultured with Wistar cardiomyocytes (Blue) or Wistar cardiomycoyte conditioned media (Green) (Uncorrected Dunn’s test; *n* = 0.5130).

By measuring the electrical properties of SHR neurons in co-culture with Wistar cardiomyocytes, we observed that co-culture reduced SHR neuron hyperexcitability (Figures 2A–C; Uncorrected Dunn’s test; *p* = 0.0345) and increased SHR neuron rheobase (Figure 2D; Uncorrected Dunn’s test; *p* = 0.0041). We hypothesized that the effect of Wistar cardiomyocytes upon SHR neuronal hyperexcitability may occur via either direct contact between neurons and cardiomyocytes, which has been previously reported to occur in co-culture (Larsen et al., 2016b) or via a releasable factor from cardiomyocytes. In the case of the latter, we theorized this would be replicable by culturing neurons with media in which cardiomyocytes had previously been cultured, thereby containing any released factors, but not the cardiomyocytes themselves. We found that this “cardiomyocyte conditioned” media reduced SHR neuronal firing (Figure 2C; Uncorrected Dunn’s test; *p* = 0.0222) and reduced SHR neuron rheobase (Figure 2D; Uncorrected Dunn’s test; *p* = 0.0219). There was no significant difference between the neuronal firing rate (Mann–Whitney test; *n* = 13, *n* = 18, *p* = 0.9929) or rheobase (Mann–Whitney test; *n* = 15, *n* = 18, *p* = 0.5796) of SHR neurons cultured with Wistar cardiomyocytes or Wistar cardiomyocyte conditioned media. Similarly, there was no significant difference between Wistar neurons alone and SHR neurons cultured in Wistar cardiomyocyte cultured media for either maximum firing rate (Mann–Whitney test; *n* = 8, *n* = 18, *p* = 0.1520) or rheobase (Mann–Whitney test; *n* = 8, *n* = 18, *p* = 0.5008). These results suggest that at least part of the effect of Wistar cardiomyocytes upon SHR neurons is mediated by one or more releasable factors.

**FIGURE 3:**
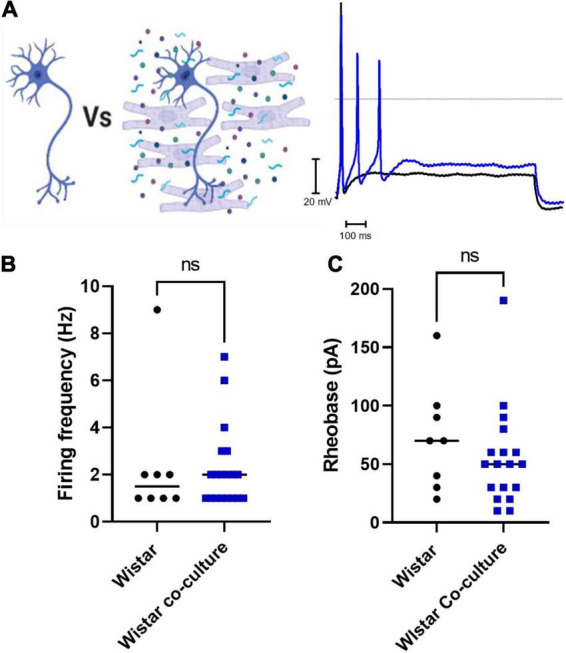
Co-culture of Wistar neurons with Wistar cardiomyocytes did not affect Wistar neuron excitability The effect of Wistar cardiomyocyte co-culture upon Wistar neuron excitability was tested here. There was no observed effect of Wistar cardiomyocyte culture upon Wistar neuron function. **(A)** Diagram demonstrating the experimental design. Here Wistar neurons (Black) were cultured with Wistar cardiomyocytes (Blue), and thus able to physically and/or chemically interact with the cardiomyocytes. Example traces of Wistar neurons cultured alone (Black) or with Wistar cardiomyocytes (Blue). **(B)** There was no significant difference in neuronal firing rate **(C)** (Mann–Whitney test; *n* = 8, *n* = 19, *p* = 0.6816) or rheobase (Mann–Whitney test; *n* = 8, *n* = 18, *p* = 0.2650) between Wistar neurons cultured alone or in co-culture (Mann–Whitney test; *n* = 8, *n* = 18, *p* = 0.2650) between Wistar neurons cultured alone or in co-culture.

Finally, we assessed whether co-culture with Wistar cardiomyocytes affected Wistar neuronal excitability. We found that there was no significant difference between Wistar neurons cultured alone or in co-culture as assessed by whole-cell current-clamp recordings of neuronal firing rate (Figures 3A,B) or neuronal rheobase (Figure 3C). Similar results were observed for Wistar neurons cultured with cardiomyocyte conditioned media (Firing rate; Mann–Whitney test; *n* = 8, *n* = 8, *p* = 0.1040; Rheobase Mann–Whitney test; *n* = 8, *n* = 8, *p* = 0.3646).

## Discussion

We report three novel findings. First, co-culturing healthy myocyte with diseased sympathetic neurons reduced pathological neuronal hyperexcitability. Secondly, when SHR neurons were perfused with conditioned media from healthy myocytes, they also exhibited a decrease in firing rate and higher rheobase, indicating that a greater current was required to induce neuronal firing. Thirdly, co-culturing healthy neurons with healthy myocytes, or alternatively, perfusing neurons with conditioned media from healthy myocytes did not alter neuronal firing, suggesting factor(s) released from healthy myocytes can significantly modulate neuronal hyperexcitability in states of sympathetic dysautonomia.

It is well established that cardiac sympathetic imbalance is a powerful driver of cardiac excitability (Nash et al., 2001) in cardiovascular disease, especially during all stages of neurogenic hypertension where it precedes the clinical signs of high blood pressure itself (Li et al., 2012; Herring et al., 2019; Hadaya and Ardell, 2020). This relationship from neuron to myocyte, has been observed in co-culture (Larsen et al., 2016b) and *in vivo* studies (Berg and Jensen, 2011). The increase in neural firing in the SHR model is driven by a plethora of ion channels, in particular downregulation of M current that restricts neural firing. Moreover, activators of M current can markedly decrease sympathetic hyperexcitability (Davis et al., 2020). The power of the neuron to drive the myocyte phenotype is reinforced by the observation that cross-culturing healthy sympathetic neurons onto diseased SHR ventricular myocytes could return the SHR myocyte cAMP response to resemble that seen in healthy heart cells. Conversely, culturing diseased neurons onto healthy heart cells could partially recapitulate the diseased myocyte response to cAMP, thus illustrating the impact of the neuron alone to drive cardiac excitability (Larsen et al., 2016b). However, the reciprocal communication from myocytes to neurons in a retrograde manner is relatively poorly studied, although changes in myocyte-vascular signaling have been implicated in sympathetic denervation (Habecker et al., 2016; Prando et al., 2018; Pianca et al., 2019). Of interest, we also observed that neurons appeared to survive longer in co-culture than in monoculture, which is similar to other anedoctal reports (Habecker et al., 2016).

### Retrograde signaling from myocyte to neuron: A modulator of neuronal excitability?

Consistent with our electrophysiological findings, emerging evidence suggests that cardiac sympathetic plasticity can be modulated by factors released from cardiac myocytes. Here Dokshokova et al. (2022) reported that nerve growth factor (NGF) from sympathetic-coupled cardiac myocytes can activate neuronal tropomyosin-receptor-kinase-A (TrkA). TrkA receptors are essential for the survival of sympathetic neurons and their distal targets *in vivo* (Fagan et al., 1996) as well as regulating the synaptic properties of the neuron (Luther and Birren, 2009). Supporting a key role for NGF, Dokshokova et al. (2022) observed that sympathetic innervated NGF-silenced cardiac myocytes appeared fragmented and had smaller TH-marked varicosities, compared with controls, which would reinforce the idea of a structural underpinning for impaired neuronal signaling from the heart itself if it is diseased. This may be particular pertinent in models of heart failure and post myocardial infarction where the primary disease is cardiac in origin (Habecker et al., 2016).

What is becoming clear is the complexity of paracrine signaling systems across different cell types in cardiac-neural tissue. Single cell sequencing studies report a diverse range of transcripts in the stellate ganglia from classical TH cells to immune cells, endothelial cells, fibroblasts and glial cells (Davis et al., 2020). When heart cells and neurons are connected, as they are *in vivo*, then the potential putative cross talks among cells is further enhanced. This is nicely illustrated in a recent study, which demonstrated sympathetic neurons can influence heart development via clock genes to regulate myocyte proliferation. Here, Tampakakis et al. (2021) inhibited NGF in vascular smooth cells, which resulted in impaired sympathetic innervation in the heart and reduced norepinephrine release. Interestingly, this was associated with proliferating cardiac myocytes and enlarged hearts that was directly related to downregulation in clock genes (*Per1/Per2*). When these circadian rhythm genes were deleted, the impaired neural phenotype and associated cardiac hypertrophy could be recapitulated, thus demonstrating the complexity of cellular interconnectivity to modulate heart function via the nervous system.

Although we demonstrate a functional relationship whereby healthy cardiomyocytes can reduce pathological sympathetic neuron hyperexcitability via a releasable factor, further work is required to elucidate the mechanism. Whilst our data shares similarities with Dokshokova et al.’s (2022) mechanism of NGF, it is unclear at this point that this is definitely the case. Importantly the field needs to establish whether putative cardiac factor(s) can prevent or reduce pathological sympathetic nerve activity *in vitro* and *in vivo* in the SHR. A clue here to the importance of this signaling pathway may reside in data from heart transplant patients, who show restoration of sympathetic innervation and improved cardiac performance during exercise (Bengel et al., 2001).

## Limitations

Our experiments suggest a mechanism by which healthy cardiomyocytes reduce SHR neuronal excitability. However, we were unable to perform the converse experiments of culturing diseased myocytes with healthy neurons because of a low SHR cardiac myocyte yield due to maternal cannibalism before post weaning of pups. This experiment could identify whether SHR cardiomyocytes could phenocopy onto healthy neurons to increase neuronal excitability by mimicking the diseased SHR neuron. Whether our results translate *in vivo* remains to be established since not all stellate neurons innervate the heart. Nevertheless, our co-culture preparation showing direct innervation of stellate neurons onto cardiac myocytes is proof-of-principle that myocytes can modulate neuronal excitability.

## Conclusions and summary

In this manuscript we have identified retrograde signaling from myocytes to sympathetic neurons, whereby SHR neurons, which are otherwise hyperexcitable exhibit electrophysiological characteristics similar to healthy Wistar neurons. We identified that this signaling was reproducible using media in which healthy Wistar cardiomyocytes had been cultured, indicating this most likely occurred via a releasable factor(s). This study therefore provides some early evidence that cardiomyocytes can regulate sympathetic electrophysiological function in states of dysautonomia.

## Data availability statement

The raw data supporting the conclusions of this article will be made available by the authors, without undue reservation.

## Ethics statement

Ethical review and approval were not required for the animal study because the keeping and use of these animals was covered by the UK Home Office Project License (PPL) of DP: 30/3031 and P707EB251. The use of animals in the experiments of this publication complied with the University of Oxford Local Ethical Guidelines and the Animals (Scientific Procedures) Act 1986 of the United Kingdom.

## Author contributions

HD helped to design and performed all the electrophysiological experiments, analyzed the data, and drafted the manuscript. DP designed the experiments and co-wrote the manuscript with HD. DL, KL, and NL contributed to cell cultures. All authors contributed to the article and approved the submitted version.
